# Pleomorphic xanthoastrocytoma with NTRK fusion presenting as spontaneous intracranial hemorrhage—case report and literature review

**DOI:** 10.3389/fped.2024.1378608

**Published:** 2024-07-23

**Authors:** Yilong Wu, Sze Jet Aw, Swati Jain, Li Yin Ooi, Enrica E. K. Tan, Kenneth T. E. Chang, Harvey J. Teo, Wan Tew Seow, Sharon Y. Y. Low

**Affiliations:** ^1^ Neurosurgical Service, KK Women’s and Children’s Hospital, Singapore, Singapore; ^2^ Department of Pathology and Laboratory Medicine, KK Women’s and Children’s Hospital, Singapore, Singapore; ^3^ Division of Neurosurgery, University Surgical Cluster, National University Health System, Singapore, Singapore; ^4^ Department of Pathology, National University Hospital, National University Health System, Singapore, Singapore; ^5^ Paediatric Haematology/Oncology Service, KK Women’s and Children’s Hospital, Singapore, Singapore; ^6^ Department of Diagnostic and Interventional Imaging, KK Women’s and Children’s Hospital, Singapore, Singapore; ^7^ Department of Neurosurgery, National Neuroscience Institute, Singapore, Singapore; ^8^ SingHealth Duke-NUS Neuroscience Academic Clinical Program, Singapore, Singapore; ^9^ SingHealth Duke-NUS Paediatrics Academic Clinical Program, Singapore, Singapore

**Keywords:** pleomorphic xanthoastrocytoma, pediatric brain tumor, spontaneous intracranial hemorrhage, gene fusion, infantile glioma

## Abstract

**Background:**

Pleomorphic xanthoastrocytoma (PXA) is a rare brain tumor that accounts for <1% of all gliomas. An in-depth understanding of PXA's molecular makeup remains a work in progress due to its limited numbers globally. Separately, spontaneous intracranial hemorrhage (pICH) is an uncommon but potentially devastating emergency in young children, often caused by vascular malformations or underlying hematological conditions. We describe an interesting case of a toddler who presented with pICH, later found to have a PXA as the underlying cause of hemorrhage. Further molecular interrogation of the tumor revealed a neurotrophic tyrosine receptor kinase (NTRK) gene fusion and CDKN2A deletion more commonly seen in infantile high-grade gliomas. The unusual clinicopathological features of this case are discussed in corroboration with published literature.

**Case presentation:**

A previously well 2-year-old male presented with acute drowsiness and symptoms of increased intracranial pressure secondary to a large right frontoparietal intracerebral hematoma. He underwent an emergency craniotomy and partial evacuation of the hematoma for lifesaving measures. Follow-up neuroimaging reported a likely right intra-axial tumor with hemorrhagic components. Histology confirmed the tumor to be a PXA (WHO 2). Additional molecular investigations showed it was negative for BRAFV600E mutation but was positive for CDKN2A homozygous deletion and a unique neurotrophic tyrosine receptor kinase (NTRK) gene fusion. The patient subsequently underwent second-stage surgery to proceed with maximal safe resection of the remnant tumor, followed by the commencement of adjuvant chemotherapy.

**Conclusion:**

To date, there are very few pediatric cases of PXA that present with spontaneous pICH and whose tumors have undergone thorough molecular testing. Our patient's journey highlights the role of a dedicated multidisciplinary neuro-oncology team to guide optimal treatment.

## Introduction

Gliomas are the most common primary central nervous system (CNS) tumors in children. Challenges to their management are often due to their broad spectrum of clinical behavior (1). In this group, pleomorphic xanthoastrocytoma (PXA) is a rare brain tumor accounting for <1% of all glial neoplasms (2). Affected patients are often adolescent or young adults who present with seizures (3). The latest World Health Organization (WHO) designates the grading of PXA as a WHO CNS Grade 2 neoplasm as it has a relatively favorable prognosis but a higher tendency to recur than other pediatric low-grade gliomas (LGG). Furthermore, up to one-third of them show features of anaplasia (WHO CNS Grade 3), characterized by increased mitotic activity and at times necrosis, which is associated with decreased overall survival (3–5). To date, an in-depth understanding of PXA's molecular makeup remains a work in progress due to its limited numbers globally (6). Separately, spontaneous intracranial hemorrhage (pICH) is an uncommon but potentially devastating emergency in the pediatric population. Specifically in young children, such cases often present with clinical and diagnostic challenges (7). Broadly speaking, pICH is predominantly associated with intracranial vascular anomalies (8). Other etiologies, such as hematological, systemic, and cardiac causes, brain tumors, and intracranial infections are comparatively less common (7). We describe an interesting case of a 2-year-old male who presented with life-threatening pICH that was eventually found to have a PXA as its underlying cause of hemorrhage. Further molecular interrogation of the tumor revealed it was negative for BRAFV600E mutation and positive for CDKN2A homozygous deletion. In addition, the tumor was found to have a neurotrophic tyrosine receptor kinase (NTRK) gene fusion more commonly seen in infantile high-grade gliomas (HGG) (9). The unusual clinicopathological features of this case are discussed in corroboration with published literature.

## Case presentation

**Figure 1:**
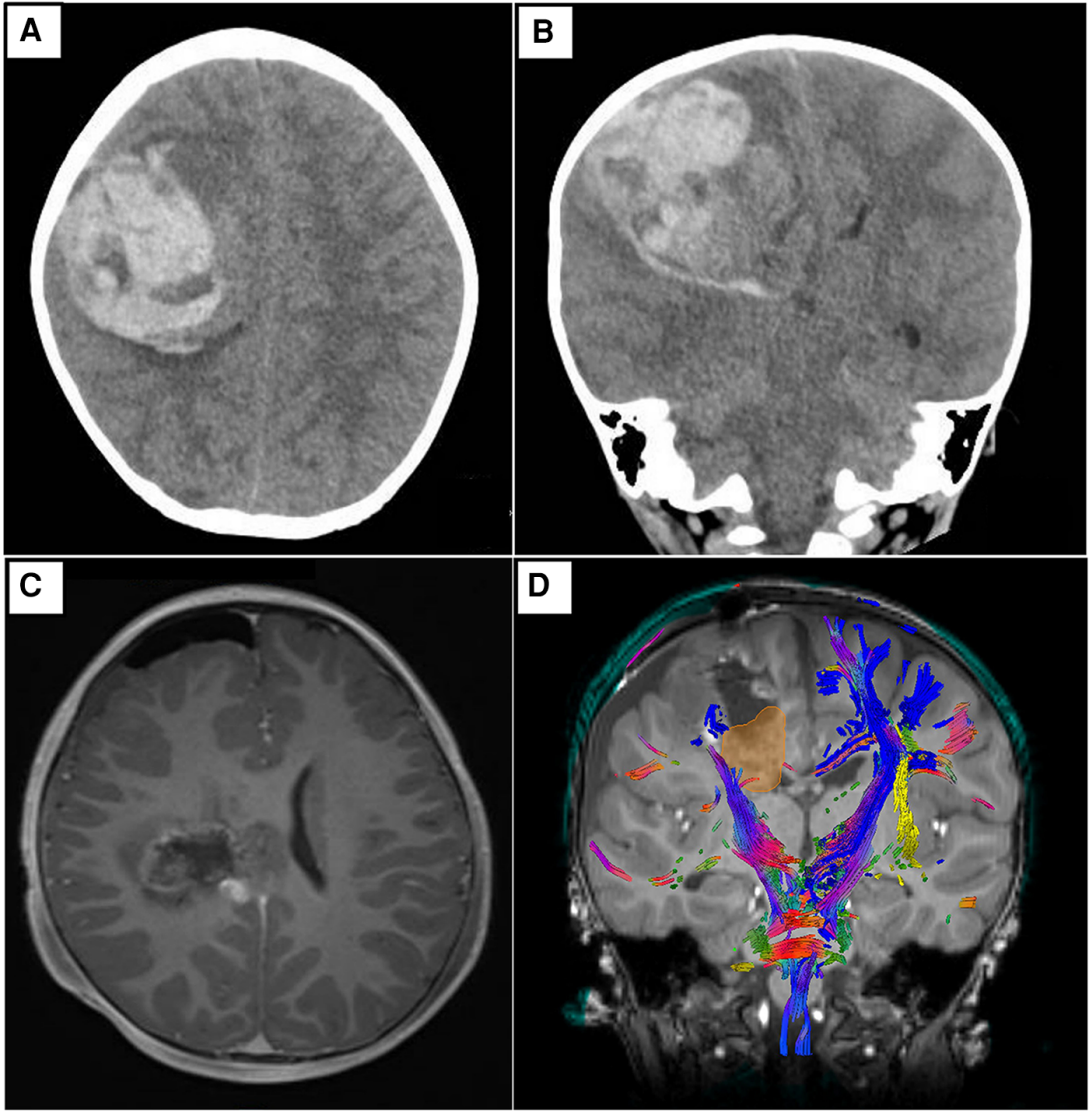
Representative non-contrast CT brain images in axial (**A**) and coronal (**B**) views. Both depict a predominantly acute intraparenchymal hematoma (6.8 × 6.4 × 5.6 cm) in the right frontoparietal region, with associated cerebral edema and mass effect. There is intraventricular extension of hemorrhage with early hydrocephalus. Subfalcine and mild right uncal herniation is seen. (**C**) Representative T1-weighted post-contrast MRI brain images in axial view. There is an irregular, ill-defined lesion with a heterogeneous signal, intermixed with blood products centered in the right frontoparietal region. The previous leftward midline shift has improved. Of note, there are foci of nodular enhancement in the periphery of the lesion that likely represent residual tumor. (**D**) Representative screenshot of relevant tractography (main corticospinal fibers in blue) in relation to the tumor (yellow-gold) from Modus Plan (Synaptive Medical, Toronto, Canada). This is a neurosurgical planning software that uses preoperative diffusion MRI data to segment out the patient's white matter tracts.

A previously well 2-year-old male presented with a 1-day onset of vomiting, drowsiness, and anisocoria. Prior to his presentation, there was no history of trauma, recent infection, or family history that could account for his clinical presentation. An urgent computed tomographic (CT) brain scan demonstrated a large, predominantly acute pICH in the right frontoparietal region with intraventricular extension, early hydrocephalus, and right uncal herniation. An emergency craniotomy with partial clot evacuation was performed for lifesaving measures. Due to the unusual diagnosis, the evacuated hematoma was sent for histopathology testing. Subsequent blood investigations to exclude underlying hematological, infective, and systemic causes were unremarkable. After the patient was medically stable, a follow-up magnetic resonance imaging (MRI) of his neuroaxis was arranged. This reported an ill-defined lesion in the right frontoparietal region that was intermixed with blood products and surrounding perilesional edema. No obvious intracranial vascular anomaly was noted. Additional diffusion tensor imaging (DTI) sequences showed that the remnant lesion had infiltrated into the right corticospinal tract. There was no radiological evidence of spinal metastases (Figure 1).

**Figure 2:**
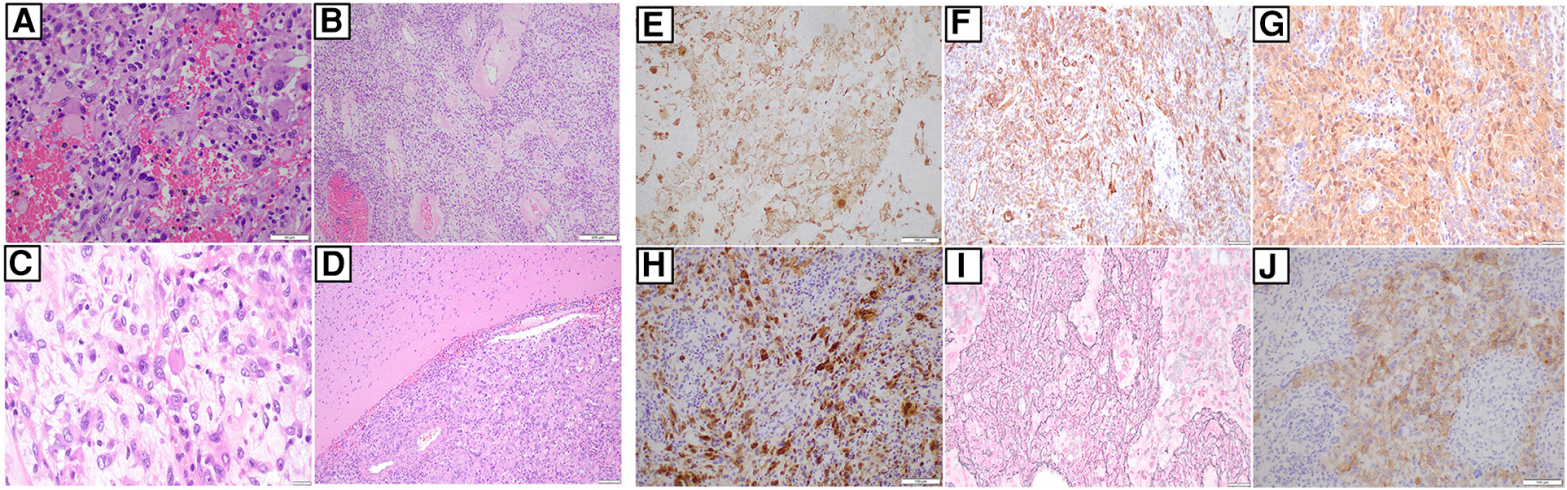
(**A**) Hematoxylin and eosin slide of tumor and it comprises of epithelioid to spindle cells, several of which feature large pleomorphic nuclei. Occasional cells show xanthomatous change. There is also intervening hemorrhage between the pleomorphic and xanthomatous cells. (40×) (**B**) Hematoxylin and eosin slide showing blood vessels with perivascular hyalinization in some areas. (10×) (**C**) This is a slide that shows the presence of eosinophilic bodies amongst the tumor cells. (40×) (**D**) Hematoxylin and eosin slide showing a well-demarcated border between the tumour and adjacent brain, without obvious infiltration. (×10). Next, immunohistochemical staining shows areas of that confirms GFAP (**E**), CD34 (**F**), S100 (**G**), and NeuN (**H**) positivity, respectively. (**I**) This is a slide that shows staining for reticulin deposited in several areas. (**J**) There is notable cytoplasmic positivity for ALK on IHC. However, follow-up molecular investigations via NGS techniques are negative for ALK (as mentioned in the main text). (All slides depicted here are 20× magnification, unless otherwise stated).

**Figure 3:**
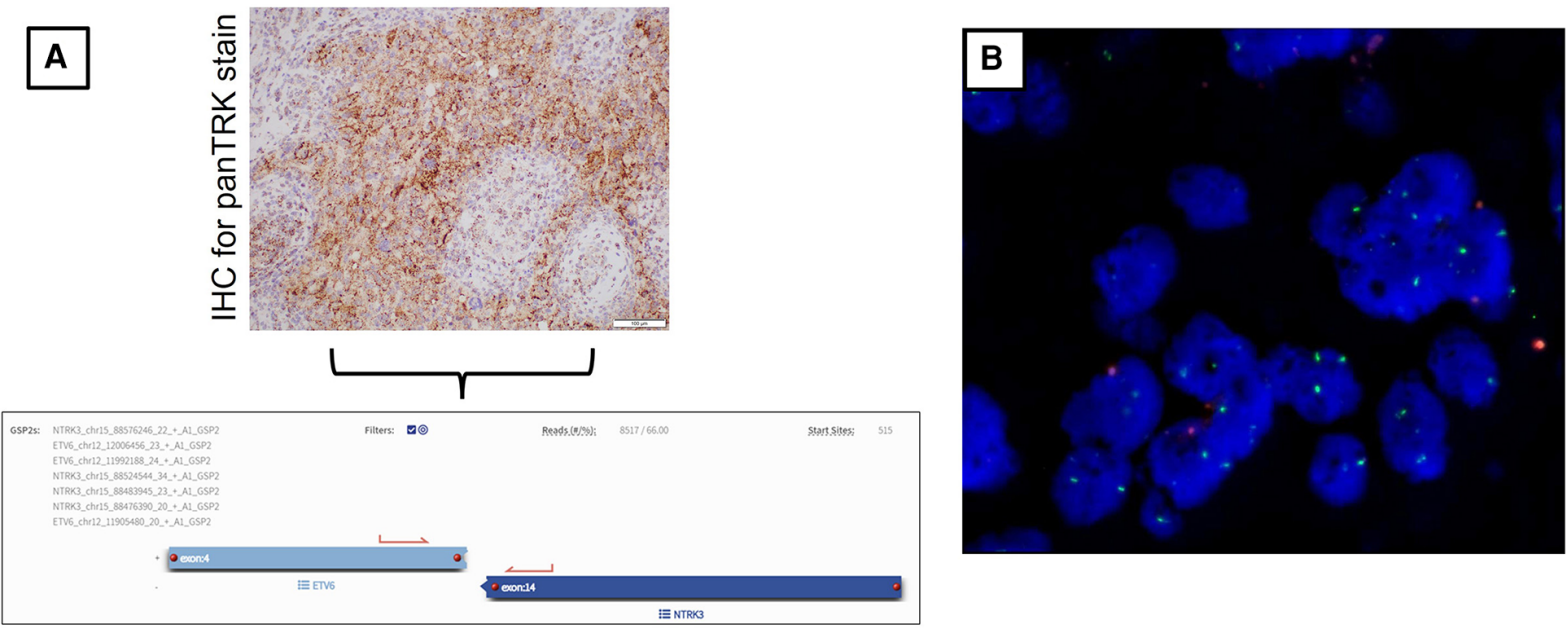
(**A**) Immunohistochemical stain for pan-TRK shows dot-like cytoplasmic positivity. (20×). This finding was followed up with the Archer FusionPlex Pan-Solid Tumour V2 (Invitae, San Francisco, CA, USA) for the patient's tumor whereby the results of the ETV6::NTRK3 fusion were found. The diagram also includes a screenshot analysis of the anchored multiplex PCR result. Here, an ETV6::NTRK3 fusion transcript with a percentage of unique reads (66%) spanning the breakpoint and supporting the event is identified. (**B**) Representative FISH image result of patient's tumor tissue. This shows the ratio of 9p21/CEP9 is 0.35, with 56% of tumor nuclei enumerated. There is no 9p21 signal, and 18% show only one copy of 9p21. Overall, these findings concur with the tumor being positive for CDKN2A deletion.

As part of our institution's integrated diagnostic workflow, each tumor's initial histopathological details are perused with the patient's clinical and radiological results. Next, the choice of molecular tests is selected based on these findings. In our case, histopathology of the evacuated hematoma reported an intrinsic glial neoplasm with a well-delineated border. Mitotic figures were present at 1 mitotic figure/mm^2^, and the Ki-67 index was up to 5%. Of note, immunohistochemistry staining for BRAF (v-raf murine sarcoma viral oncogene homolog B1) V600E was negative. Other illustrative details of the findings are described in Figures 2 and 3. Cumulative features at this juncture resulted in the preliminary diagnosis of a WHO CNS Grade 2 PXA. Due to noticeable positivity for both ALK and pan-TRK (i.e., Figures 2E, F) during the workup, additional testing via the Archer FusionPlex Pan-Solid Tumour V2 (Invitae, San Francisco, CA, USA) was performed. This is a commercially available high-throughput next-generation sequencing (NGS) panel that identifies gene translocations and internal tandem duplications across solid tumors and sarcomas in 137 genes. This investigation demonstrated the presence of an ETV6::NTRK gene fusion (Figure 3A). The tumor was then further interrogated by the Ampliseq Childhood Cancer Panel (Illumina, San Diego, CA, USA), another NGS-based targeted gene panel, which also confirmed the presence of the ETV6::NTRK fusion. In addition, the results showed the absence of reportable single-nucleotide variants (in particular, BRAFV600E) or copy number variants. Interestingly, we noted that no sequence alteration or fusion was detected in the ALK gene for both NGS techniques. In this setting, the decision was made to concur with the NGS findings. However, a homozygous deletion of cyclin-dependent kinase inhibitor 2A (CDKN2A) was simultaneously detected. In view of this latter finding, a fluorescence *in situ* hybridization (FISH) test for the CDKN2A gene was ordered, which established its deletion in the tumor. Put together, the eventual diagnosis of a “WHO CNS Grade 2 PXA with an ETV6::NTRK fusion” was made. The patient underwent a second-stage resection to remove the remnant hemorrhagic tumor approximately 2 weeks after his initial surgery. Intraoperatively, the decision was made to leave a small sliver of tumor that infiltrated into the corticospinal tract to avoid neurological injury. Otherwise, he recovered well to a full Glasgow Coma Scale with no residual neurological deficit. The tumor specimen submitted from the second surgery was histopathologically similar to the patient's initial surgery.

The case was presented at a neuro-oncology multidisciplinary tumor (MDT) board. The MDT consisted of specialists from pediatric oncology, neuroradiology, pathology, neurosurgery, radiation oncology, and allied health teams. Specifically for this patient, the following were discussed: firstly, there was still a remnant tumor in an eloquent region of the brain; next, the Ki-67 index was 5%; and lastly, the molecular findings of an NTRK gene fusion included the lack of BRAFV600E mutation and CDKN2A homozygous deletion. The consensus was to manage the diagnosis as per an infantile HGG. A referral to the cancer genetics team was arranged. Despite much discussion during the consultation, the patient's parents declined to pursue the recommended germline testing for the patient. Subsequently, the patient was commenced on a carboplatin-based regimen of chemotherapy (10), with the option of an NTRK inhibitor in the event of relapse. In view of his young age, the role of proton beam therapy was to be held off as long as possible. Concurrently, a referral to the cancer genetics team was made for the patient to discuss the role of germline testing. However, his parents declined to proceed after the consultation. At approximately 10 months after his initial presentation, the patient remains clinically well without radiological evidence of tumor recurrence. To date, he is noted to be active and has no neurological deficit or significant developmental delay.

## Discussion

### Pleomorphic xanthoastrocytoma: current understanding

PXA was first described in 1979 as a distinct type of glioma that is postulated to have originated from subpial astrocytes (11). Presently, Kepes et al's (11) original description of a supratentorial astrocytoma that has a superficial cortical location and unique histological features, such as marked cellular pleomorphism, rich reticulin network, and prominent lipid-laden glial cells, is still relevant. PXA tends to be found in the supratentorial region, particularly in the temporal lobe. At times, both the leptomeninges and superficial regions of the cerebrum are anatomically involved (4). Occasionally, infratentorial cases have been described in the literature (4, 12). On neuroimaging, PXA is usually peripherally located and frequently cystic, involving the cerebral cortex and overlying leptomeninges (3). Demographically, there are conflicting reports on gender predilection with some papers reporting equal occurrence in both sexes and others claiming a slight male preponderance (13). Studies report that PXA is commonly diagnosed in the second decade of life (13–15). Prevalence in the infantile HGG group (here, referring to children younger than 3–5 years old) is infrequent (16). Regarding clinical presentation, affected patients tend to present with seizures (3, 17). Neurosurgical intervention, especially total excision of the tumor has been proven to be associated with good survival outcomes (18). Although PXA is reputed to have a relatively favorable prognosis, it has an increased risk of recurrence in comparison to other pediatric LGGs. This slightly more aggressive behavior has led to its designation as either a WHO Grade 2 or 3 CNS neoplasm (3). Existing literature shows that up to one-third of PXA tumors demonstrate anaplastic characteristics such as higher mitotic activity and necrosis, whereby both are associated with decreased overall survival (4–6). This particular subgroup is termed PXA WHO Grade 3. These tumors are more aggressive and have been reported to spread via cerebrospinal fluid (CSF), often in the setting of disease recurrence or malignant transformation (19, 20). For the PXA WHO Grade 2 tumors, the Ki-67 labeling index is generally <1%, whereas up to 15% has been reported in their WHO Grade 3 counterparts (3, 13, 21).

### Clinical relevance of molecular information and gene fusions

The advent of technological advancements has provided valuable insights into the molecular profiles of pediatric gliomas. We are now aware that a significant proportion of PXA cases harbor a BRAF V600E gene mutation and or CDKN2A gene alterations (4, 22, 23). In addition, an in-depth study by Mistry et al. (23), which focused on the subset of pediatric LGG that transformed into secondary HGG, demonstrates that BRAFV600E mutations and CDKN2A deletions constitute a clinically distinct subtype of secondary HGG. Essentially, they report that BRAF and CDKN2A gene alterations are less common in the pediatric LGG cohort that do not show malignant transformation and patients with BRAF mutations had longer latency to secondary HGG (23). Next, it is noteworthy to mention the presence of CDKN2A deletion in our patient's tumor. CDKN2A is a tumor suppressor gene that encodes the p16INK4a protein and serves as an inhibitor of cell cycle progression. Molecular insights have revealed that it is a major target of mutation in many human cancers (24). Previous studies have shown that the CDKN2A homozygous deletion is an important prognostic factor for survival outcomes of IDH-mutant glioma patients regardless of histology grading (25). For instance, homozygous deletion involving the CDKN2A locus found in adult WHO Grade 3 oligodendrogliomas has been linked to lower survival, regardless of microvascular proliferation with or without necrosis (3, 26). Furthermore, in histologically lower-grade adult gliomas, CDKN2A homozygous deletion is associated with a more aggressive clinical course and is a molecular marker of Grade 4 status in the latest WHO classification (3, 27). Interestingly, a recent study of 67 PXA tumors reports up to 94% of them have pre-existing CDKN2A/B deletions. However, further analyses demonstrate this genomic alteration is not associated with overall survival (5). Instead, WHO grading for PXA is a stronger predictor of survival in the study cohort (5). Overall, molecular information for tumors is important due to paradigm shifts toward targeted therapies for challenging cancers, especially brain tumors (28). For example, the preliminary evidence for BRAF inhibitors for disease control obtained by BRAF inhibitors in tumors harboring the BRAF V600E mutation has been optimistic (29, 30). Similarly, the identification of cyclin-dependent kinase inhibitor (CDK) gene alterations has paved the way for the development of CDK-related therapeutics against various malignancies, including gliomas (31–33).

Following that, the remaining PXA cases without BRAFV600E (including our patient) have been reported to harbor RTK (receptor tyrosine kinase) gene fusions rather than MAPK(mitogen-activated protein kinase) alterations (34). At the time of this writing, there is only one other pediatric PXA case in the literature that reports an NTRK fusion (35). Broadly speaking, gene fusions are pathognomonic mutations resulting from a hybrid of two or more coding regulatory DNA sequences between genes due to genomic rearrangements from translocations, deletions, duplications, or inversions (36–38). Gene fusions are clinically relevant because they provide important information on tumorigenesis that pave the way for the development of targeted therapies for patients with specific fusions (39). In recent years, transcriptomic analyses have uncovered a subset of glioma patients carrying gene fusions (39). For affected patients, it has been reported that gene fusions can occur up to 30%–50% in high-grade gliomas (36, 37, 40). To date, collaborative genomic studies have demonstrated that infantile HGGs comprise molecularly distinct subgroups that are characterized by gene fusions (9, 41). Examples include the NTRK fusions that have been described as oncogenic drivers in several human tumors, including pediatric gliomas (42). The NTRK family comprises NTRK1, NTRK2, and NTRK3 that encode the neurotrophin receptors tropomyosin-related kinase (TRK) groups such as TrkA, TrkB, and TrkC. Gene fusions involving the NTRK family are one of the most common mechanisms of oncogenic TRK activation (43). This is clinically relevant because NTRK fusions represent a pharmacologically targetable genomic alteration (35). Therefore, potential NTRK-targeted treatment offers hope for very young brain tumor patients with limited treatment options. To date, NTRK inhibitors are currently being offered in clinical trials for primary CNS tumors with promising results (44). Here, the option of an NTRK inhibitor for our patient is feasible if conventional treatment methods fail.

Put together, a summary of our patient's pertinent molecular findings includes the following: the lack of BRAFV600E mutation, the presence of CDKN2A homozygous deletion, and an ETV6::NTRK gene fusion. Based on the current disease understanding of this rare brain tumor, it may be difficult to draw definitive conclusions in regard to the molecular results' prognostic impact on our patient. However, we are aware that PXA tumors frequently recur and are associated with decreased survival compared with other LGGs in children and young adults (3). Furthermore, malignant progression is known to be more common in PXA in comparison to other RAS/MAPK-driven LGGs (3, 45).

### Neoplasm-related intracranial hemorrhage in children: an overview

In view of our patient's unusual clinical presentation, an overview of non-traumatic intracranial hemorrhagic in children is included in the following discussion. Non-traumatic pediatric intracranial hemorrhage (pICH) is defined as a brain parenchymal bleed with or without intraventricular extension occurring between 29 days and 18 years of age (46, 47). Typically, the incidence of pICH is cited to be extremely low in children. The exact incidence is unclear owing to current evidence relying on mostly case reports and small case series (35, 47, 48). Despite its rarity, pICH is an important cause of death and irreversible neurological injury (8). Delayed diagnosis is common because very young children have difficulties in accurately communicating complaints, and the hemorrhage can be manifested by non-specific symptoms such as irritability, somnolence, or headache. In addition, lateralizing neurological symptoms are reported less frequently in the pediatric population as compared to their adult counterparts (7, 49). Regardless, management should be prompt and thorough diagnostic workup to guide optimal clinical treatment (46, 47).

Of note, the pICH etiological spectrum differs from the adult population and is largely dominated by cerebral vascular lesions, hematological disorders, neoplasia, and systemic diseases. In addition, the literature shows that pICH can be secondary to various tumor types, including benign to malignant neoplasms (47). Erosion of cortical artery by the tumor, thin-walled tumor vessel, or unknown microvascular anomaly within the tumor have been postulated to be contributing factors for the bleeding episode (18, 50, 51). Specific to PXA, there have been only 3 other pediatric cases presenting as intracranial bleeds in the literature (Table 1).

**Table 1:** Summary of pediatric cases of PXA presenting as intracranial hemorrhage.

Case number/reference	Age (years)/gender	Tumor location	Staged surgery (yes/no)	Extent of resection	Histology/Ki-67 or MIB-1	Presence of BRAFv600e mutation (yes/no)	Additional molecular investigations
1 (Wind, 2009)	5/female	Left temporal	Yes	GTR	PXA (Ki-67, 2–3%)	Unknown	NIL
2 (Takamine, 2019)	11/female	Right temporal	No	GTR	PXA (Ki-67, 2%)	No	NIL
3 (Pehlivan, 2020)	6/male	Left frontoparietal	No	GTR	PXA (“low”)	No	Yes, NGS panel
4. Our case	2/male	Right parietal	Yes	NTR	PXA (Ki-67, 5%)	No	Yes, NGS panel and FISH

## Conclusion

The unique features of this case highlight challenges faced by various specialist teams and emphasize the importance of an integrated multidisciplinary approach to patient care. Although vascular anomalies constitute most of the underlying causes of pICH, clinicians need to be mindful to include as a differential diagnosis of brain tumors during an acute presentation. From the tumor diagnosis perspective, the role of additional molecular studies has enabled valuable information to guide adjuvant treatment. This case report adds to the limited pool of medical literature for this rare primary brain tumor. Moving forward, we advocate collaborative clinical and in-depth molecular studies at an international level.

## Data Availability

The original contributions presented in the study are included in the article/Supplementary Material; further inquiries can be directed to the corresponding author.
